# Skin tolerant inactivation of multiresistant pathogens using far-UVC LEDs

**DOI:** 10.1038/s41598-021-94070-2

**Published:** 2021-07-19

**Authors:** Johannes Glaab, Neysha Lobo-Ploch, Hyun Kyong Cho, Thomas Filler, Heiko Gundlach, Martin Guttmann, Sylvia Hagedorn, Silke B. Lohan, Frank Mehnke, Johannes Schleusener, Claudia Sicher, Luca Sulmoni, Tim Wernicke, Lucas Wittenbecher, Ulrike Woggon, Paula Zwicker, Axel Kramer, Martina C. Meinke, Michael Kneissl, Markus Weyers, Ulrike Winterwerber, Sven Einfeldt

**Affiliations:** 1 Ferdinand-Braun-Institut gGmbH, Leibniz-Institut für Höchstfrequenztechnik, Gustav-Kirchhoff-Str. 4, 12489 Berlin, Germany; 2 Institut für Festkörperphysik, Technische Universität Berlin, Hardenbergstr. 36, 10623 Berlin, Germany; 3 Institut für Optik und Atomare Physik, Technische Universität Berlin, Straße des 17. Juni 135, 10623 Berlin, Germany; 4 Center of Experimental and Applied Cutaneous Physiology, Department of Dermatology, Venerology and Allergology, Charité - Universitätsmedizin Berlin, Corporate Member of Freie Universität Berlin and Humboldt-Universität zu Berlin, Charitéplatz 1, 10117 Berlin, Germany; 5 Institut für Hygiene und Umweltmedizin, Universitätsmedizin Greifswald, Ferdinand-Sauerbruch-Straße, 17475 Greifswald, Germany; 6 Georgia Institute of Technology, Atlanta, GA USA

**Keywords:** Disease prevention, Biomedical engineering, Inorganic LEDs, Infectious diseases

## Abstract

Multiresistant pathogens such as methicillin-resistant *Staphylococcus aureus* (MRSA) cause serious postoperative infections. A skin tolerant far-UVC (< 240 nm) irradiation system for their inactivation is presented here. It uses UVC LEDs in combination with a spectral filter and provides a peak wavelength of 233 nm, with a full width at half maximum of 12 nm, and an irradiance of 44 µW/cm^2^. MRSA bacteria in different concentrations on blood agar plates were inactivated with irradiation doses in the range of 15–40 mJ/cm^2^. Porcine skin irradiated with a dose of 40 mJ/cm^2^ at 233 nm showed only 3.7% CPD and 2.3% 6-4PP DNA damage. Corresponding irradiation at 254 nm caused 11–14 times higher damage. Thus, the skin damage caused by the disinfectant doses is so small that it can be expected to be compensated by the skin's natural repair mechanisms. LED-based far-UVC lamps could therefore soon be used in everyday clinical practice to eradicate multiresistant pathogens directly on humans.

## Introduction

The increasing resistance of bacteria to antibiotics is one of the major challenges facing mankind in the area of global health^1^. Currently, about 700,000 patients worldwide die every year from an infection with multidrug resistant organisms (MROs). The trend is rising: The number is estimated at ten million for the year 2050^2^. This means that more people would then die of MROs than of cancer. Furthermore, the current worldwide pandemic with the SARS-CoV-2 virus also calls for a method to eradicate viruses efficiently and sustainably. This is preferably achieved by a physical method that non-selectively and irreversibly inactivates all microorganisms so that no resistance can be developed. Such a method must consequently attack the microorganism at a vital yet non-specific point.

UVC radiation can inactivate microorganisms and viruses by triggering photochemical reactions in the DNA or RNA. As a result, they can no longer replicate and their potential pathogenic efficacy is thus inhibited^3–6^. This type of disinfection is currently used for drinking water^7^, and also for cleaning surfaces^8^ and air^9^. The required dose for the inactivation of microorganisms and viruses depends strongly on the individual species, their environment and the wavelength of the radiation^10–17^. Generally, the UV absorption spectrum of DNA and RNA exhibits a maximum at ~ 265 nm and a minimum around ~ 240 nm and increases again for shorter wavelengths. The main advantage of using far-UVC radiation (< 240 nm) instead of near-UVC radiation (250–280 nm) for the inactivation is its lower penetration depth in the skin^18^. Far-UVC radiation is mainly absorbed in the uppermost, non-living cornified layer of the skin and potentially causes little damage to the living cells underneath, as previously shown in mice^19^. This gives rise to the vision of antisepsis of skin surfaces by direct UVC irradiation without serious damage to health. This vision is supported by numerous studies conducted in recent years on the skin tolerance of far-UVC radiation^20–22^.

Investigations on a possible in vivo antisepsis with far-UVC radiation have so far mostly been limited to the use of excimer lamps with Kr-Cl emitting at 222 nm, or with Kr-Br emitting at 207 nm^5^. In the clinical environment directly on humans they are of limited use: Such lamps are bulky, fragile, emit considerable heat and are operated at high voltages. The same applies to the application of lasers^23^. Only recently, LEDs with a peak emission wavelength in the range of 230–240 nm and milliwatt output powers have been demonstrated for the first time^24,25^ after detailed optimization of the design and manufacturing conditions^26–29^. In the long run, far-UVC LEDs should be superior to excimer lamps in many ways. Due to their compact design, low-voltage operation and flexible adjustable wavelength, many of the antisepsis applications in humans could greatly benefit from or become possible by using far-UVC LEDs. This applies to wound antisepsis during surgery, the decolonization of methicillin-susceptible *Staphylococcus aureus* (MSSA) in the nasal vestibule as the main source of such infections^30^ or the inactivation of corona viruses directly in their habitat of the mucous membrane in the throat^31^. However, in view of strong absorption of far-UVC radiation, it is unclear to what extent it can reach microorganisms in such an environment at all. In any case, corresponding experimental studies with far-UVC LEDs are, however, completely lacking so far.

With this study we are taking the first step towards a practical medical application of far-UVC radiation on humans. For this purpose, we designed and manufactured a far-UVC irradiation system based on LEDs. A dense array of 120 LEDs, which is additionally equipped with a spectral filter, delivers spectrally pure far-UVC radiation with a peak wavelength of 233 nm and negligible power components at > 240 nm. The suitability of the irradiation system for in vivo antisepsis applications is demonstrated by the successful inactivation of Methicillin-resistant *Staphylococcus aureus* (MRSA) and the proof that the radiation causes hardly any damage to porcine skin.

## Results

### Design and performance of the far-UVC LED irradiation system

**Figure 1:**
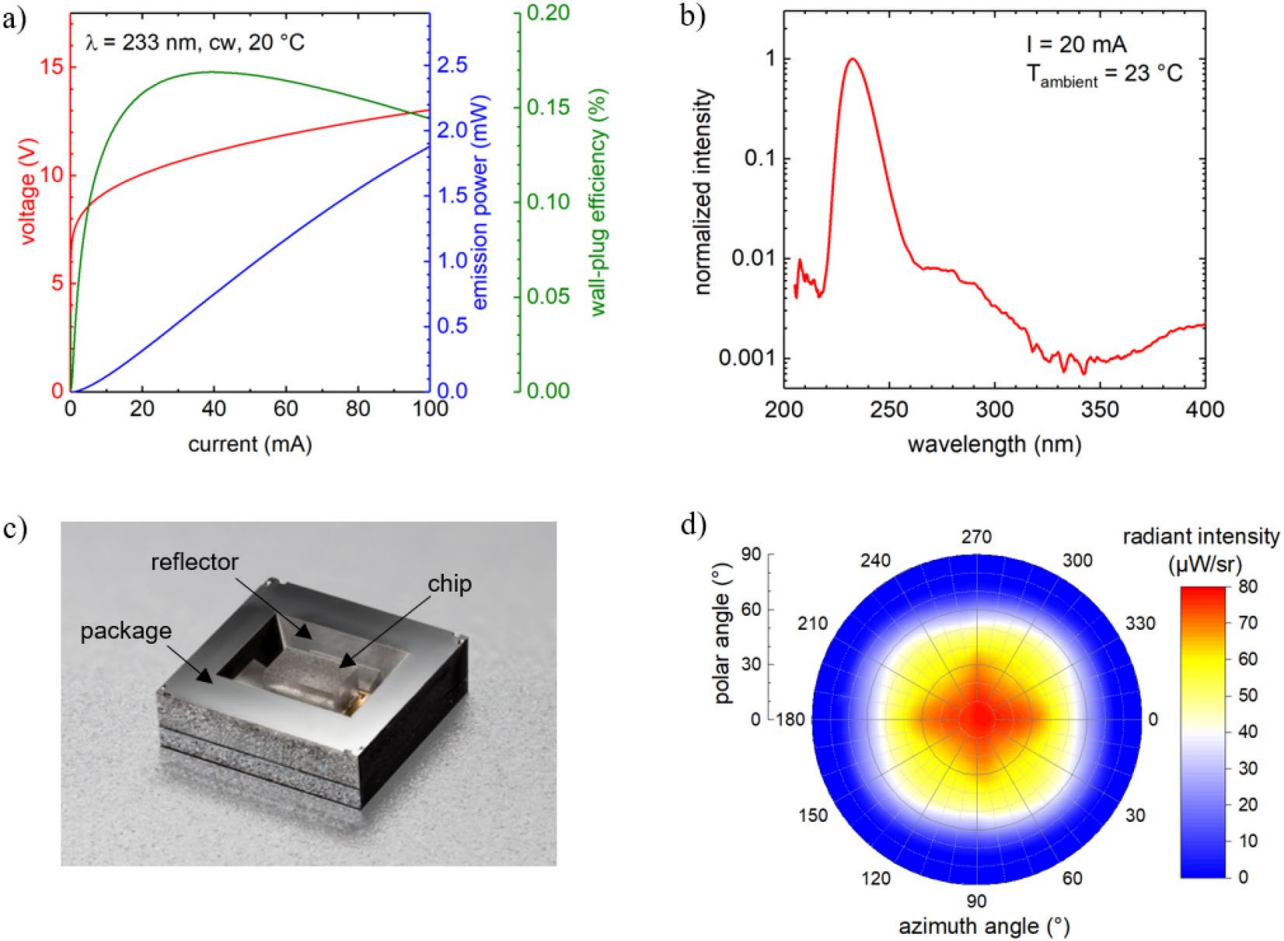
(**a**) Voltage, wall-plug efficiency and optical power as a function of current of a typical flip-chip mounted 233 nm LED used in the irradiation module. (**b**) Emission spectrum of the LED operated at 20 mA. (**c**) Image of a 233 nm LED flip-chip mounted on a Si-cavity surface-mounted device package (footprint = 3.5 mm × 3.5 mm). (**d**) Far-field radiation distribution measured of a typical 233 nm LED operated at 20 mA.

The key components of the irradiation system are far-UVC LEDs, based on AlGaN semiconductor heterostructures grown by metalorganic vapor phase epitaxy on sapphire substrates (see Methods). At an operating current of 100 mA and a heat sink temperature of 20 °C, the operation voltage of the LEDs is 13 V with a maximum emission power of (1.9 ± 0.3) mW. This corresponds to an external quantum efficiency of (0.36 ± 0.07) % and wall-plug efficiency (WPE) of (0.14 ± 0.02) % (Fig. 1a). The LEDs have a moderately narrowband emission with a peak wavelength of 233 nm and a full width at half maximum of 12 nm (Fig. 1b). In addition, a weak shoulder around 300 nm and a small side peak at about 400 nm are observed, caused by deep level transitions at Mg defects^27^. The flip-chip packaged far-UVC LEDs typically have a viewing angle of 148°. The far field pattern exhibits four local maxima at 40° inclined to the chip normal caused by nearly 50% of the emitted light being emitted via the side surfaces of the LED chip^25^. To obtain a more directional radiation, a specially designed silicon-based surface mounted device package^32^, with an integrated aluminum reflector was used for the packaging of the LEDs (Fig. 1c). In addition, a Zener diode, for protection against damage from electrostatic discharge, is monolithically integrated in the base plate of the package by ion implantation. In this way, the distance between the reflector and the LED chip could be minimized and shadowing effects were avoided. The 54.7° angled reflector surfaces deflect the light emitted from the side surfaces of the LED chip towards the front resulting in a smaller viewing angle of 110° and a twofold increase in the radiance along the surface normal (Fig. 1d).

**Figure 2:**
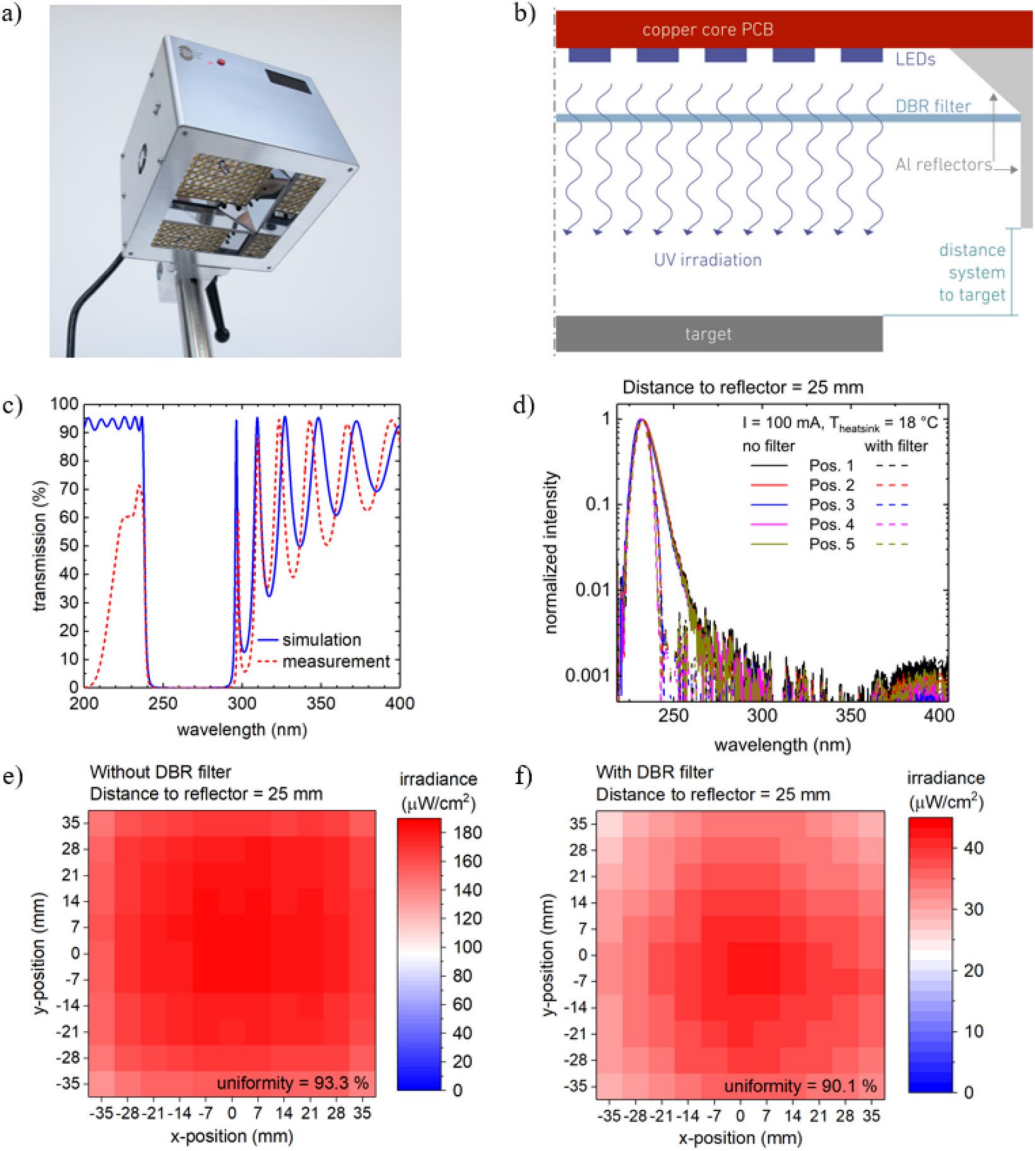
(**a**) Picture of the completely assembled far-UVC LED irradiation system where the radiation unit with the LEDs and the vertical and slanted reflectors are visible. (**b**) Half of a cross section schematic of the optical components of the far-UVC LED radiation unit. (**c**) Simulated (blue curve, solid line) and measured (red curve, dashed line) transmission spectra of the DBR filter under normal incidence. (**d**) Emission spectra measured at the maximum operation current of the LEDs (100 mA), at a distance of 25 mm from the lower edge of the vertical reflector and at five different positions (center (Pos. 1) and four corners of the LED array (Pos. 2–5)). (**e**) and (**f**) The irradiance distribution of the far-UVC LED irradiation system on a 70 mm × 70 mm target area measured (**e**) without and (**f**) with the DBR filter at the maximum operation current of the LEDs (100 mA) and at a distance of 25 mm from the lower edge of the system. In both cases the uniformity (defined as 100% − (standard deviation/mean value of the irradiance)) is > 90%.

Despite huge improvements in recent years, the WPE, temperature sensitivity, spectral purity and lifetime of far-UVC LEDs are still inferior to those of near-UVC LEDs. In view of this, special measures have been taken regarding the cooling, radiation guidance and replaceability of the LEDs in the irradiation system, which is shown in Fig. 2(a) and (b). Furthermore, excellent uniformity of the emission spectra and irradiance over the target area were considered to be of key importance for the irradiation studies of skin samples and surfaces doped with viruses or bacteria. 120 far-UVC LEDs on an area of 80 mm × 80 mm combined with a two-stage aluminum reflector (Fig. 2a and b) were incorporated in the irradiation system to obtain a uniform irradiance distribution over a target area of 70 mm × 70 mm at a distance of 25 mm from the system. Due to the large number of LEDs and the low WPE of the devices, approximately 150 W of heat is generated in the system during operation. It is known that the optical power of the far-UVC LEDs, operated at 100 mA, reduces to a quarter when the temperature is increased from 20 to 80 °C^25^. This heat generated during operation is not only detrimental to the performance of the LEDs but could also heat up the surrounding air which in turn could damage the irradiated samples. To overcome this challenge, a water-cooled, copper-based heat sink, maintained at a constant temperature of 18 °C was developed for the radiation unit. Using this cooling system, the temperature of the active region of the LEDs is maintained at 34 °C at the maximum operation current of 100 mA (see supplementary material).

The stability of the LED emission is essential to the irradiation studies. In the case of the far-UVC LEDs, the emission power of the LEDs decreases very rapidly at the beginning^33^. At a constant current of 100 mA corresponding to a nominal current density of 67 A/cm^2^ in the active region of the device, the emission power of the LEDs decreases to 30% of its initial value within the first 100 h of operation^25^. Hence, to ensure stable operation of the irradiation system, the complete system was burned-in, at 100 mA, for 48 h. After this burn-in, a < 0.17%/h change in the irradiance is expected.

Although the LEDs show a single peak emission at 233 nm and FWHM of 12 nm, the weak parasitic luminescence, at wavelengths > 240 nm, could potentially cause damage to the skin as it penetrates much deeper in the skin. A distributed Bragg reflector (DBR), based on quarter wavelength stacks of HfO_2_ and SiO_2_ layers, was designed to reflect the emission between 240 and 300 nm and transmit the peak emission wavelength at 233 nm (see Methods). The design was optimized for normal incidence with nominal transmission values of 50% and 0.1% at 235 nm and 244 nm, respectively. At normal incidence, the filter shows a transmission of over 60% around 230 nm and below 0.05% above 245 nm (Fig. 2c). However, the LEDs exhibit a wide far-field of the radiated power with significant contributions emitted at angles > 30° inclined to the chip normal (Fig. 1d). Since the DBR stop band shifts to shorter wavelength with increasing angle of incidence, e.g. by 15 nm for 40°, the limitation to normal incidence considerably underestimates the real reduction of the total optical power by the filter. Calculations based on the far-field pattern, LED spectra and DBR transmission at different angles suggest a reduction of the total optical power down to 20–30%.

The emission spectra of the irradiation system, measured across the target area, are uniform both without and with the DBR filter (Fig. 2d). When using the filter, the long wavelength part of the spectra is cut off. The spectral power density at 240 nm is only 10% of that at 233 nm and decreases further at longer wavelengths, while leaving the peak wavelength almost unchanged. A weak parasitic luminescence (three orders of magnitude lower than the peak intensity) can be observed between 360 and 450 nm both with or without the use of the filter. In addition, an excellent uniformity is obtained for the irradiance distribution across the target area (Fig. 2e and f). Uniformity factors of 93% and 90% are obtained without and with the DBR filter, respectively. The irradiance decreases nearly linearly when increasing the distance from the system from 3 to 75 mm without negatively affecting the uniformity factor. The mean value of the irradiance of the system at a distance of 25 mm from the system is 170 µW/cm^2^ and reduces to 36 µW/cm^2^ when the filter is introduced.

### Inactivation of multiresistant pathogens and skin tolerance investigations

**Figure 3:**
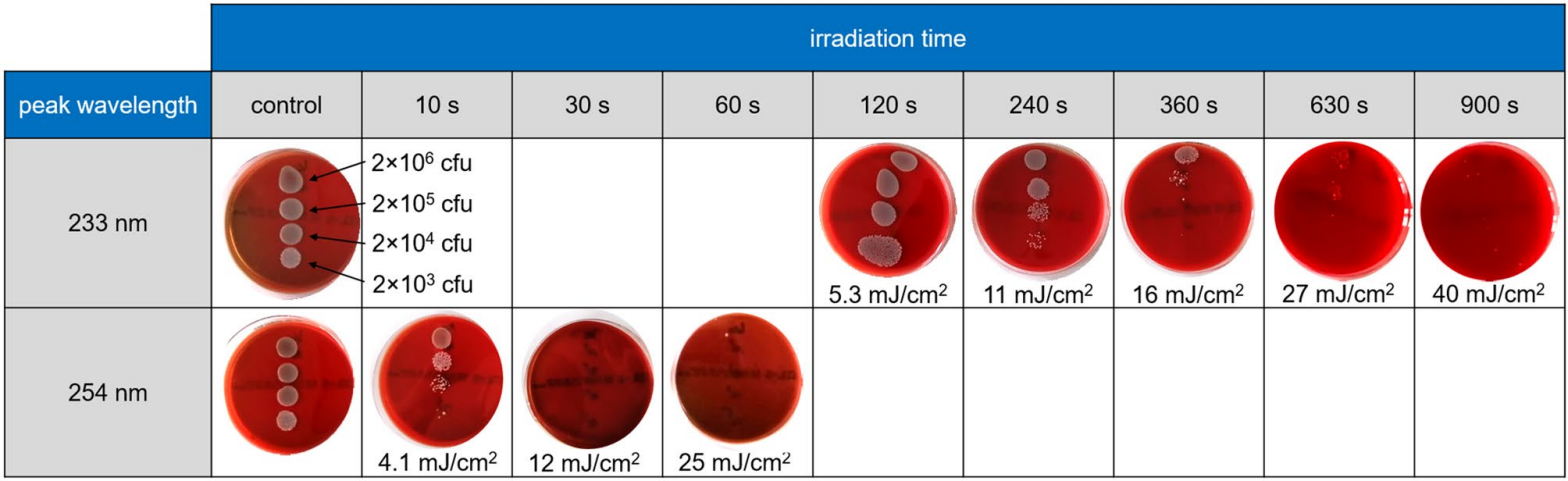
Columbia blood agar plates inoculated with different concentrations of bacterial suspension (MRSA, DSM 11822) without and with UVC irradiation for different times using either far-UVC (233 nm) or near-UVC (254 nm) radiation and after incubation for 24 h. The numbers below the plates indicate the applied UV dose.

MRSA was chosen as a model organism for the inactivation studies using far-UVC radiation because although there is a great clinical need to inactivate them, the process of doing so is difficult. A spot test as qualitative verification of bacterial inactivation was performed on Columbia blood agar plates using *Staphylococcus aureus* (DSM 11822). The far-UVC irradiation system, with the filter, was used with an irradiance of 44 µW/cm^2^. In addition, UVC radiation from a low-pressure mercury gas-discharge lamp emitting at a peak wavelength of 254 nm with an irradiance of 440 µW/cm^2^ was applied as positive control. As can be seen in Fig. 3, bacteria growth is visible on control plates that have not been irradiated and bacteria growth was inhibited on irradiated plates. More precisely, at 254 nm a UV dose of 4.4 mJ/cm^2^ is sufficient for a visible reduction of bacterial regrowth at small bacteria concentrations (approximate 3 lg-levels). A nearly complete inactivation of the bacteria with a reduction factor of about 6 lg-levels was achieved by a UV dose of 13 mJ/cm^2^. At 233 nm, an approximately 20-fold higher UV dose of 11 mJ/cm^2^ to 16 mJ/cm^2^ is effective in inhibiting growth of bacteria at lower concentrations (2 × 10^3^ cfu/spot to 2 × 10^4^ cfu/spot) displaying reduction factors of 3–4 lg-levels. Higher bacteria concentrations were nearly completely inactivated by applying 233 nm UV doses of 27 mJ/cm^2^ to 40 mJ/cm^2^ (4–6 lg levels).

It is commonly known that UVC radiation with wavelengths < 280 nm is very harmful to human skin^34^. However, the shorter the wavelength of the UV radiation, the higher the absorption in the stratum corneum. Consequently, less radiation reaches the cell nuclei in the keratinocytes in the viable epidermis and DNA damage in the skin is reduced. To prove this assumption, the far-UVC irradiation system has been tested in comparison to a low-pressure mercury gas-discharge lamp emitting at a wavelength of 254 nm with an irradiance of 550 µW/cm^2^ as a positive control on porcine skin which is a suitable skin model for human skin^35^. The far-UVC irradiation experiments were performed with and without the filter using an irradiation dose of 40 mJ/cm^2^ (46 µW/cm^2^ for about 14.5 min with filter and 178 µW/cm^2^ for 3.75 min without filter) which is able to inactivate MRSA as explained above.

**Figure 4:**
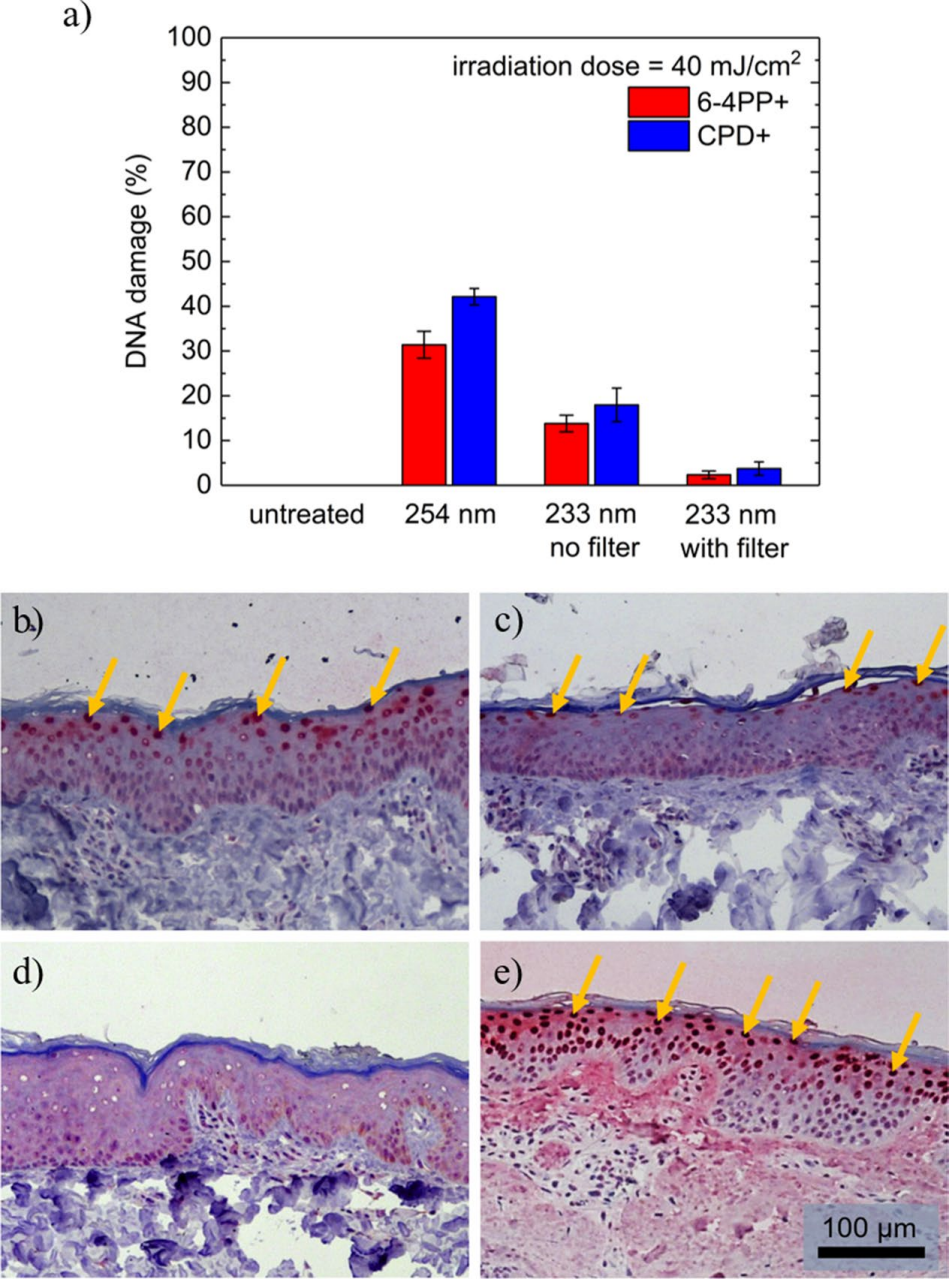
(**a**) DNA damage for irradiated porcine skin using 254 nm and 233 nm light sources with 40 mJ/cm^2^. Untreated skin served as control. The mean values of the epidermal DNA damage in % are calculated from at least three experiments and the error bars show the standard error of the mean. (**b**)–(**e**) Histologic images HE and CPD stained porcine skin after irradiation using the far-UVC LED irradiation system without (**c**) and with filter (**d**), in comparison, untreated skin (**e**) and skin after irradiation with near-UVC radiation at 254 nm (**f**). Arrows mark CPD positive cells. The scale bar is 100 µm.

Figure 4 (a) shows the results of the skin damage investigations. The cyclobutane pyrimidine dimers (CPD) and pyrimidine (6–4) pyrimidone photoproducts (6-4PP) damage is presented in percentage of epidermal cells with DNA damage to the total amount of cells in the microscopic images. CPD stained histologic images of one such sample can be seen in Fig. 4(b–e). The 254 nm UV radiation led to a high amount of (42 ± 2) % CPD and (31 ± 3) % 6-4PP DNA damage. 6-4PP stained histologic images are shown in the supplementary material (Fig. S1). In comparison, untreated skin did not show any damage. Irradiation with 233 nm UV radiation without the filter induced (18.0 ± 3.5) % of CPD damage and (13.8 ± 1.9)% of 6-4PP damage. When using the filter these numbers are further reduced to (3.7 ± 1.5) % of CPD damage and (2.3 ± 0.8) % of 6-4PP damage. These results show that the far-UVC radiation at 233 nm strongly reduces the DNA damage compared to near-UVC radiation at 254 nm, which is usually used for the decontamination of surfaces. The filter which significantly suppresses spectral contributions of the LEDs at wavelengths above 240 nm is able to further reduce DNA-damage by a factor of 5. An important fact is that unlike the use of near-UVC radiation at 254 nm, the DNA damage caused by far-UVC radiation at 233 nm appears only superficially below the horny layer of the skin (stratum corneum, SC) in the upper half of the epidermis and does not reach the sensitive basal cells (Fig. 4b). The CPD damage induced by 254 nm irradiation occurs partly until the basal layer. The basal cells in particular must be protected from damage, as the renewal process of the epidermis starts from there every 28 days. The studies will be extended to human skin in the future. Human skin includes melanin and could have a different SC thickness compared to porcine skin. A temperature increase of the skin during irradiation was neither observed at irradiation with 233 nm, nor at 254 nm.

## Discussion

The LED-based far-UVC irradiation system has been demonstrated to be capable of inactivating multiresistant microorganisms without causing significant skin damage. MRSA as a model organism for MROs in concentrations of 2 × 10^3^ to 2 × 10^6^ cfu on blood agar plates was inactivated within 6 to 15 min. These times are sufficiently short to allow the system to be used in a clinical setting.

Observed difference in the MRSA inactivation effectiveness using UVC radiation with a wavelength of either 233 nm or 254 nm might be due to the difference in the absorbance of UV radiation by DNA depending on the radiation wavelength^36^. Another factor that influences the efficacy is that on agar plates the bacterial suspension partially permeates into the agar. However, the shorter the wavelength of radiation, the shorter is its penetration depth. Therefore, the radiation might not reach the bacteria hidden in deeper areas of the agar. Additionally, these bacteria then are protected by sheep erythrocytes, absorbing the radiation by its proteins and DNA^37^. Hence, a higher dose is most likely needed for complete inactivation of the bacteria when using radiation at 233 nm instead of 254 nm. In addition, similar investigations on another strain of MRSA, *Staphylococcus aureus* DSM 18827, showed complete inactivation with a 233 nm UV dose of 40 mJ/cm^2^.

These results on the MRSA inactivation on blood agar plates using far-UVC radiation emitted by LEDs are very promising. However, further in vitro experiments have to be performed before a far-UVC LED irradiation system should be used for human skin antisepsis. Particularly, tests on carrier discs are necessary, where the use of different soil loads such as urea, proteins (albumin) or fatty acids, is possible and no permeation will take place. This allows a better imitation of the conditions on the human skin to exclude any influence of the environment on the antibacterial effectiveness.

The applied far-UVC radiation doses are skin compatible: At a radiation duration of 14.5 min, damage to porcine skin was only 3.7% (CPD) and 2.3% (6-4PP), respectively. This is a factor of 11 to 14 less damage than compared to the application of an equivalent near-UVC radiation at 254 nm and actually so low that apoptosis^38^ and the natural enzymatic repair mechanisms of the skin^39^ can compensate for the induced damage. This has been shown using 254 nm irradiation on hairless mice, where the initial 37% CPD damage decreased to 13% within 24 h^19^. On which dynamics the repair mechanisms occur and if a threshold of CPD damage is necessary to trigger such processes, as suggested for UVA irradiation^40^, will be subject of future investigations, where DNA damage needs to be determined at varying time points subsequent to irradiation.

The CPD damage is in comparable magnitude to recently published data using 222 nm irradiation. Narita et al.^41^ found no CPD damage in hairless mice after applying a dose of 150 mJ/cm^2^. In a recent in vivo study on human skin, Fukui et al.^21^, found a slight but significantly increased amount of CPD damage compared to non-irradiated skin, which was determined by an ELISA essay. Due to the use of different skin models, a direct comparison between 233 and 222 nm radiation has to be evaluated in future studies on more viable skin models, which are more metabolically active.

The basic principle of skin safe far-UVC antisepsis has been reported before^17–22^. In this respect, the results of this work confirm the previous findings. However, all previous work was carried out using excimer lamps, while far-UVC LEDs with a different wavelength were used here for the first time. Only far-UVC LEDs might enable the inactivation of MROs in clinically relevant contexts, such as the decolonization of the nasal vestibule. In this work, the feasibility of such an approach has been successfully demonstrated. Nevertheless, the development of appropriate LED irradiation systems is still in its infancy. Shorter irradiation times are desirable which require more efficient and longer lasting LEDs. This entails above all optimization on the side of the LED—in the design and fabrication of the semiconductor layer structure and the chip as well as in the packaging. The necessary performance improvements of the far-UVC LEDs can be expected, considering the success story of blue LEDs in the lighting sector, but also the progress made for near-UVC LEDs over the last few years. We are therefore convinced that in a few years far-UVC LEDs will outperform their lamp competitors in certain aspects and make their way into clinical in vivo antisepsis in humans. This will be a major step towards solving global health problems due to MROs.

## Methods

### Growth of the far-UVC LED heterostructures

A 720 nm thick AlN base layer was grown in an 11 × 2″ Aixtron Aix2400G3-HT planetary metal–organic vapor phase epitaxy (MOVPE) reactor on 2″ diameter c-plane sapphire substrates (offcut 0.1° towards an m-direction) using trimethylaluminum and ammonia as sources and hydrogen as carrier gas. The growth pressure, temperature and growth rate were 50 hPa, 1180 °C and 1.5 µm/h, respectively^42^. During growth, the V/III ratio was reduced from 450 to 30 under constant TMAl flow of 350 µmol/min. Parallel ridges of 2.0 µm width, 3.5 µm pitch and 1.3 µm height were processed into these AlN/sapphire templates by means of photolithography and inductive coupled plasma (ICP) etching. For this a hard mask of 1 µm thick SiN_x_ deposited by plasma-enhanced chemical vapor deposition and patterned by SF6-based ICP etching was used. The mask was then transferred into the AlN/sapphire templates by BCl_3_-based ICP etching. The patterned wafers were overgrown (ELO, epitaxial lateral overgrowth) by 5.2 µm AlN in the MOVPE reactor as described above^43^.

The UV LED heterostructure was grown in a 3 × 2″ closed-coupled showerhead reactor using trimethylaluminium, trimethylgallium, triethylgallium, silane, bis-cyclopentadienyl magnesium and ammonia as precursors as well as hydrogen and nitrogen as carrier gases. The LED heterostructure consists of a 400 nm AlN buffer layer, a 25 nm transition layer from AlN to Al_0.87_Ga_0.13_ N, 100 nm undoped Al_0.87_Ga_0.13_ N, a 1.2 μm Al_0.87_Ga_0.13_ N:Si current spreading layer optimized for high transparency and low resistivity, a 40 nm thick Al_0.83_Ga_0.17_ N:Si first barrier, a threefold Al_0.72_Ga_0.28_ N(1 nm)/Al_0.83_Ga_0.17_ N:Si(5 nm, 2.5 nm central delta Si-doping) multiple quantum well active region, a 6 nm AlN electron blocking layer to minimize electron leakage but facilitate hole injection, a 14-fold Al_0.8_Ga_0.2_ N:Mg(0.9 nm)/ Al_0.7_Ga_0.3_ N:Mg(0.9 nm) short period superlattice, a 15-fold Al_0.37_Ga_0.63_ N:Mg(2.5 nm)/ Al_0.2_Ga_0.8_ N:Mg(2.5 nm) superlattice, and a 40 nm GaN:Mg contact layer. The Mg doped layers were activated by thermal annealing in the MOVPE reactor at 830 °C under nitrogen atmosphere for 25 min and subsequently in an oven at 600 °C under oxygen for 20 min.

### Processing of the far-UVC LEDs

The chip processing sequence for the fabrication of the n-contacts involved the exposure of the buried n-type current spreading layer by ICP etching using a pure Cl_2_ chemistry, the thermal deposition of a V(15 nm)/Al(90 nm)/Ni(20 nm)/Au(30 nm) metal stack and its annealing at 800 °C under nitrogen for 40 s. The p-contact was fabricated by electron-beam deposition of Pt(30 nm) which is annealed at 500 °C under nitrogen for 5 min. The metal contacts were enhancement by electron-beam deposition of a Ti(30 nm)/Pt(40 nm)/Au(500 nm)/Ti(30 nm) metal stack. 600 nm SiN_x_, deposited by plasma-enhanced chemical vapor deposition, was used for passivation and its structuring was achieved by inductively coupled plasma etching using CHF_3_. Finally, the bonding pads consisting of a Ti(30 nm)/Pt(40 nm)/Au(475 nm)/Ti(30 nm)/Pt(120 nm)/Au(300 nm) metal stack was deposited using electron-beam deposition. The wafers were diced into individual 0.66 mm × 1.06 mm dies using an internally focused laser beam scriber at a wavelength of 533 nm and a chip breaker. The LED chip design consists of interdigitated p- and n-contact fingers with a total emitting area of ~ 0.15 mm^2^.

### Packaging of the far-UVC LED chips

The far-UVC LED chips were flip-chip mounted on 3.5 mm × 3.5 mm surface-mounted device (SMD) Si-based packages (developed by CiS Forschungsinstitut für Mikrosensorik GmbH) with a thermal conductivity of 150 W/(m·K) using AuSn solder paste^32^. The thickness of the Au–Sn layer was determined to be 15 µm.

### Electro-optical characterization of the far-UVC LEDs

The emission spectrum and optical power of the far-UVC LEDs were measured using a radiometric and wavelength calibrated compact spectrometer (StellarNet EPP2000-UV–VIS) and a calibrated UV-enhanced Si-photodiode (Hamamatsu S2281-01) with an active area of 1 cm^2^, respectively. The far-field emission distribution of the far-UVC LEDs was determined by measuring the radiant intensity in angular steps of 5° on an automated two axes rotation stage using a calibrated UV-enhanced Si-photodiode (Thorlabs FDS010) with an active area of 0.8 mm^2^ at a distance of 37 mm from the LED. In addition, the total optical power of the far-UVC LEDs was determined by spatial integration of the far-field emission distribution over the entire forward hemisphere. All measurements were performed under continuous wave (cw) operation at room temperature without active cooling.

### Optical simulations of far-UVC LEDs and irradiation system

The required ray-file of the far-UVC LED for the optical simulation of the irradiation system was generated using a self-developed Monte-Carlo ray-tracing simulation program based on Mathematica 10 and PureBasic 5.71. The ray-tracing simulations consider the complex refractive indices of AlGaN^44–46^, sapphire^47^ and contact metals^48^ at 233 nm, as well as the absorption within the multiple quantum well region (10^3^ cm^−1^)^49^, the n-AlGaN (50 cm^−1^) and p-(Al)GaN (1.7 × 10^5^ cm^−1^) heterostructure layers^50^. In addition, the radiation pattern of the active region was determined by the degree of polarization ((TE − TM)/(TE + TM) =  − 0.4)^51^. The light scattering at the ELO AlN/sapphire interface and the rough sapphire sidewalls of the chip, as well as the reflection at the Al-reflector of the Si-based SMD package were taken into account^32,51^. Surface roughness was described by the distribution of corresponding microscopic slope angles.

Ray-tracing simulations of the far-UVC irradiation module using the software ZEMAX-EE^52^ were used to optimize the irradiance uniformity and to achieve a maximum irradiance. The model consists of 120 LEDs with an individual optical power of 0.5 mW. The reflector areas (slanted and vertical) were defined with a reflectance of 70% as measured using the same aluminum-based reflector material (MIRO, ALANOD GMBH & CO. KG, Ennepetal) that was used in the far-UVC irradiation system. The fully absorbing target area with a size of 100 mm × 100 mm was placed at a distance of 50 mm to the lower edge of the reflectors.

### Simulation and fabrication of DBR filter

The DBR filter consists of 18 pairs of HfO_2_ and SiO_2_ layers deposited on a 1 mm thick and 100 mm × 100 mm large fused silica substrate. The optimal layer thicknesses were determined by simulations using a self-written software based on the transfer-matrix method. The individual layers thicknesses were varied slightly at random to suppress high order interference effects oscillations around 233 nm. The dielectric layers were deposited using a "Syrus 710 pro" from Bühler with two electron beam evaporators and ion support (APS pro). The reduction of the total emission power of the far-UVC LEDs by the DBR filter was estimated using the measured far-field emission distribution together with the angular dependent transmission spectrum of the DBR filter.

### Electro-optical characterization of far-UVC irradiation system

The irradiance distribution of the system was measured with a radiometrically calibrated photodetector (UV-Surface-USB, Sglux GmbH). The emission spectrum was measured with a spectrally calibrated compact UV–VIS spectrometer (USB4000, Ocean Optics, Inc). Both detectors were attached to a motorized xyz-stage to scan the radiation at different positions and distances.

### Investigation of MRSA by UVC irradiation

Methicillin resistant strains of the Gram-positive bacterium *Staphylococcus aureus* (DSM 11822 and DSM 18827) were used to test the antimicrobial efficacy of far-UVC radiation using a qualitative spot test. First, cryopreserved bacteria were inoculated on Columbia blood agar (Becton Dickinson GmbH, Heidelberg, Germany) and incubated at 37 °C for 24 h. For the second subculture, one colony was plated on tryptic soy agar (TSA) and incubated again at 37 °C for 24 h. Next, the microorganisms were harvested by rinsing the agar plate with 2 ml phosphate buffered saline (PBS). The bacteria were pelleted by centrifugation for 1 min at 2500 × *g* and washed 3 times with 1 ml PBS. Each washing step was followed by centrifugation for 1 min at 2500 × *g*. The resulting pellet was resuspended in CASO bouillon, and a final suspension of 1–3 × 10^8^ colony forming units (cfu)/ml was produced by adjusting the optical density (OD) at 620 nm to 0.1–0.15. Afterwards, a dilution series in CASO bouillon was produced. Four different dilutions of the bacterial suspension (1 × 10^8^, 1 × 10^7^, 1 × 10^6^, 1 × 10^5^) were used for the spot tests: 20 µl of the suspension were dropped onto the agar plate and dried for 30 min at room temperature in a laminar airflow chamber, resulting in 2 × 10^3^–2 × 10^6^ cfu/spot. DIN 14561, for testing chemical disinfectants and antiseptics in the medical area using the quantitative carrier test, defines a reduction factor of five lg-levels (99.999%) as necessary for adequate reduction of microorganisms. Therefore, the various bacterial suspensions with different concentrations were used to ensure the detection of the requested reduction factor.

The bactericidal reduction factor (RF) can be calculated according to the following equation, where *n*_*c*_ is the number of cfu on control specimen without irradiation and *n*_*p*_ is the number of cfu on irradiated test specimen:

$$ RF = \log_{10} (n_{c} ) - \log_{10} (n_{p} ). $$

A calculation using the presented spot test is not possible, however, an estimation of lg levels is possible, since the initial number of bacteria in each spot was quantified.

The intensity of 44 µW/cm^2^ of the 233 nm far-UVC irradiation system equipped with the DBR filter was checked before the experiments at the system in a steady state using the UV radiometer SXL55 with a SiC UVC sensor (Sglux GmbH). The same procedure was conducted with a 254 nm wavelength mercury gas-discharge lamp (Sglux GmbH) used as positive control. The 254 nm emitter was dimmed to 440 µW/cm^2^. The agar plates were irradiated for different durations (10 s–15 min), resulting in irradiation doses of 4.4–40 mJ/cm^2^. After irradiation, the plates were incubated at 37 °C for 24 h for the growth of viable bacteria.

### Investigation of skin tolerance to UVC irradiation

Porcine ears were freshly obtained from a local butcher and the experiments were performed within 48 h after slaughter. Hair was removed from the skin without impacting the stratum corneum using scissors and the skin was dermatomized to 300 µm thickness. For UV irradiation experiments, sections of 2 cm × 2 cm were placed on a wet paper tissue to prevent drying out. The irradiation experiments were performed in standardized laboratory conditions at 21 °C. After irradiation, 4 mm punch biopsies were taken and fixated in neutral buffered 4% formalin solution (Sigma # HT501128-4L). Subsequently, the formalin-fixed samples were dewatered and embedded in paraffin (Histosec, Merck Millipore) overnight. Paraffin blocks were prepared per sample and 1–2 µm thick sections were cut from these blocks. Sections were dewaxed and histochemically stained with hematoxylin (Merck Millipore) and eosin (Sigma Aldrich) for evaluation of histomorphology or subjected to a heat-induced epitope retrieval step prior to immunohistochemistry. DNA damage was immunohistochemically detected via expression of cyclobutane pyrimidine dimers (CPD) and pyrimidine (6–4) pyrimidone photoproducts (6-4PP) (see Fig. 4b-e). For this purpose, sections were incubated with either anti-6-4PP (clone 64 M-2, Cosmo Bio) or anti-CPD (clone TDM-2, Cosmo Bio). Dako REAL Detection System, Alkaline Phosphatase/RED, Rabbit/Mouse (Agilent Technologies) was employed for the detection of 6-4PP and CPD. Nuclei were counterstained with hematoxylin (Merck Millipore) and slides coverslipped with Kaiser’s glycerol gelatine (Merck Millipore). Negative controls were performed by omitting the primary antibody. Histologic images of the stained skin sections were acquired using an AxioImager Z1 microscope (Carl Zeiss MicroImaging, Inc.). The percentage of positively stained cells within the epidermis was evaluated in a blinded manner.

## Supplementary Information

Supplementary Information.
