# Author Correction: Skin tolerant inactivation of multiresistant pathogens using far-UVC LEDs

**DOI:** 10.1038/s41598-022-11796-3

**Published:** 2022-05-11

**Authors:** Johannes Glaab, Neysha Lobo‑Ploch, Hyun Kyong Cho, Thomas Filler, Heiko Gundlach, Martin Guttmann, Sylvia Hagedorn, Silke B. Lohan, Frank Mehnke, Johannes Schleusener, Claudia Sicher, Luca Sulmoni, Tim Wernicke, Lucas Wittenbecher, Ulrike Woggon, Paula Zwicker, Axel Kramer, Martina C. Meinke, Michael Kneissl, Markus Weyers, Ulrike Winterwerber, Sven Einfeldt

**Affiliations:** 1 Ferdinand-Braun-Institut gGmbH, Leibniz-Institut für Höchstfrequenztechnik, Gustav‑Kirchhoff‑Str. 4, 12489 Berlin, Germany; 2 Institut für Festkörperphysik, Technische Universität Berlin, Hardenbergstr. 36, 10623 Berlin, Germany; 3 Institut für Optik und Atomare Physik, Technische Universität Berlin, Straße des 17. Juni 135, 10623 Berlin, Germany; 4 Center of Experimental and Applied Cutaneous Physiology, Department of Dermatology, Venerology and Allergology, Charité ‑ Universitätsmedizin Berlin, Corporate Member of Freie Universität Berlin and Humboldt-Universität zu Berlin, Charitéplatz 1, 10117 Berlin, Germany; 5 Institut für Hygiene und Umweltmedizin, Universitätsmedizin Greifswald, Ferdin and-Sauerbruch-Straße, 17475 Greifswald, Germany; 6 Georgia Institute of Technology, Atlanta, GA USA

Correction to: *Scientific Reports* https://doi.org/10.1038/s41598-021-94070-2, published online 19 July 2021

The original version of this Article contained an error in the reported measurement values of the irradiance and irradiation doses of the low-pressure mercury gas-discharge lamp emitting at a peak wavelength of 254 nm.

As a result, in the Abstract

“Corresponding irradiation at 254 nm caused 15–30 times higher damage.”

now reads:

“Corresponding irradiation at 254 nm caused 11–14 times higher damage.”

Additionally, in the Results section, under the subheading ‘Inactivation of multiresistant pathogens and skin tolerance investigations’,

“In addition, UVC radiation from a low-pressure mercury gas-discharge lamp emitting at a peak wavelength of 254 nm with an irradiance of 50 µW/cm^2^ was applied as positive control.”

now reads:

“In addition, UVC radiation from a low-pressure mercury gas-discharge lamp emitting at a peak wavelength of 254 nm with an irradiance of 440 µW/cm^2^ was applied as positive control.”

“More precisely, at 254 nm a UV dose of 0.5 mJ/cm^2^ is sufficient for a visible reduction of bacterial regrowth at small bacteria concentrations (approximate 3 lg-levels).

now reads:

“More precisely, at 254 nm a UV dose of 4.4 mJ/cm^2^ is sufficient for a visible reduction of bacterial regrowth at small bacteria concentrations (approximate 3 lg-levels).”

“A nearly complete inactivation of the bacteria with a reduction factor of about 6 lg-levels was achieved by a UV dose of 1.5 mJ/cm^2^.”

now reads:

“A nearly complete inactivation of the bacteria with a reduction factor of about 6 lg-levels was achieved by a UV dose of 13 mJ/cm^2^.”

“To prove this assumption, the far-UVC irradiation system has been tested in comparison to a low-pressure mercury gas-discharge lamp emitting at a wavelength of 254 nm with an irradiance of 50 µW/cm^2^ as a positive control on porcine skin which is a suitable skin model for human skin^35^.”

now reads:

“To prove this assumption, the far-UVC irradiation system has been tested in comparison to a low-pressure mercury gas-discharge lamp emitting at a wavelength of 254 nm with an irradiance of 550 µW/cm^2^ as a positive control on porcine skin which is a suitable skin model for human skin^35^.”

“The 254 nm UV radiation led to a high amount of (58 ± 11) % CPD and (69 ± 12) % 6-4PP DNA damage.”

now reads:

“The 254 nm UV radiation led to a high amount of (42 ± 2) % CPD and (31 ± 3) % 6-4PP DNA damage.”

“The CPD damage induced by 254 nm irradiation occurs throughout the entire epidermis.”

now reads:

“The CPD damage induced by 254 nm irradiation occurs partly until the basal layer.”

In the Discussion section,

“This is a factor of 15 to 30 less damage than compared to the application of an equivalent near-UVC radiation at 254 nm and actually so low that apoptosis^38^ and the natural enzymatic repair mechanisms of the skin^39^ can compensate for the induced damage.”

now reads:

“This is a factor of 11 to 14 less damage than compared to the application of an equivalent near-UVC radiation at 254 nm and actually so low that apoptosis^38^ and the natural enzymatic repair mechanisms of the skin^39^ can compensate for the induced damage.”

In the Methods section, under the subheading ‘Investigation of MRSA by UVC irradiation’,

“The 254 nm emitter was dimmed to 50 µW/cm^2^. The agar plates were irradiated for different durations (10 s–15 min), resulting in irradiation doses of 0.5–40 mJ/cm^2^.”

now reads:

“The 254 nm emitter was dimmed to 440 µW/cm^2^. The agar plates were irradiated for different durations (10 s–15 min), resulting in irradiation doses of 4.4–40 mJ/cm^2^.”

Furthermore, this Article contained an error in Figure 3 where irradiance values were incorrect. The original Figure 3 and accompanying legend appear above.

**Figure 3:**
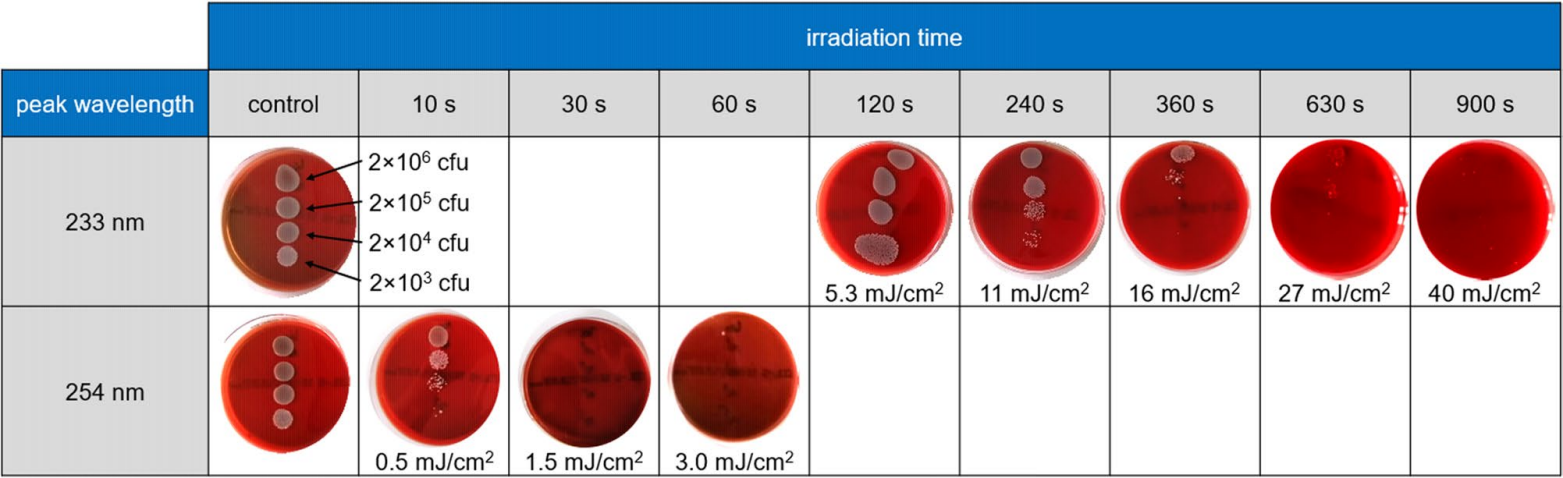
Columbia blood agar plates inoculated with different concentrations of bacterial suspension (MRSA, DSM 11822) without and with UVC irradiation for different times using either far-UVC (233 nm) or near-UVC (254 nm) radiation and after incubation for 24 h. The numbers below the plates indicate the applied UV dose.

This Article also contained an error in Figure 4 where the experiments with the low-pressure mercury gas-discharge lamp were incorrect. The original Figure 4 and accompanying legend appear above.

**Figure 4:**
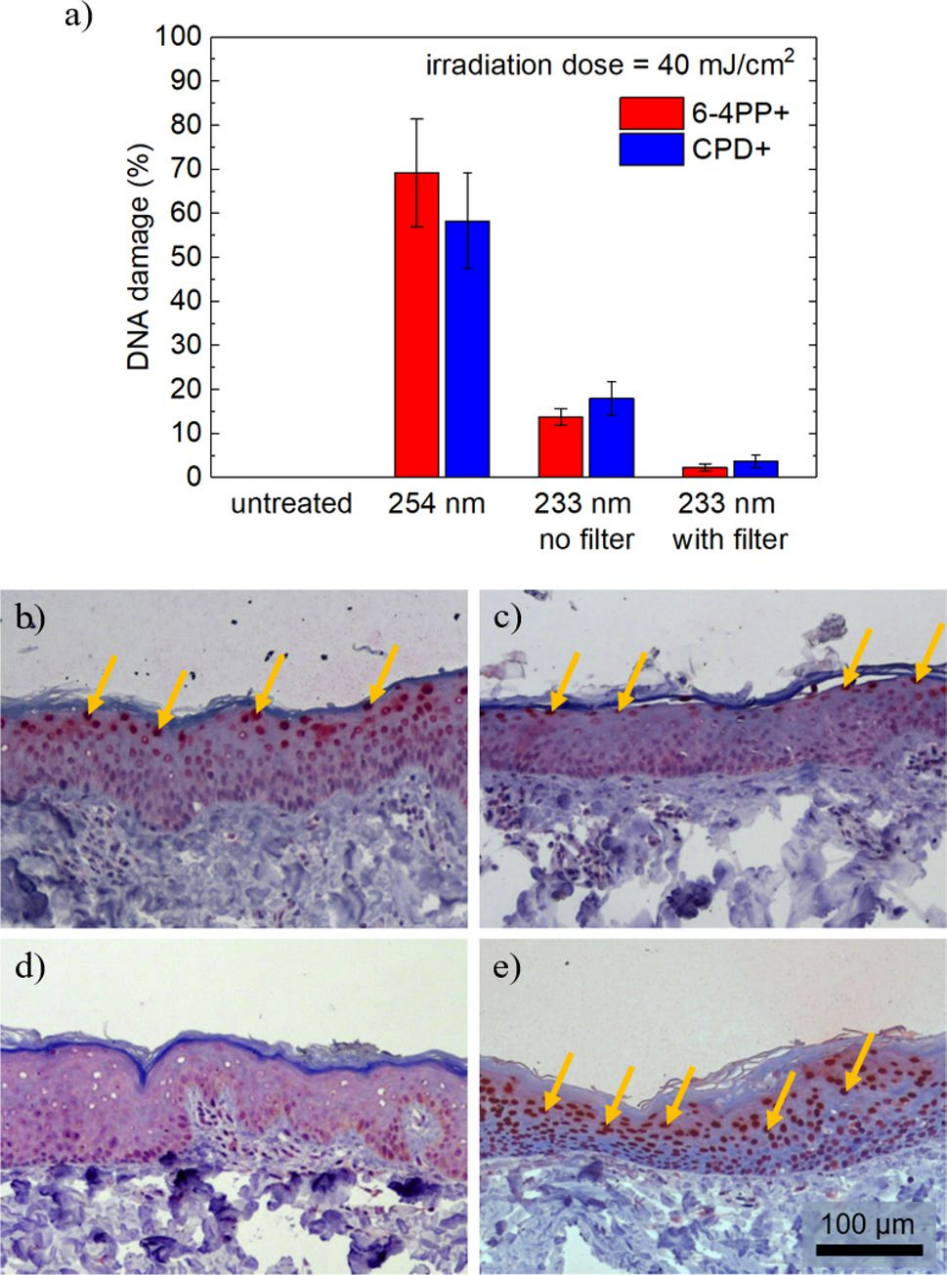
(**a**) DNA damage for irradiated porcine skin using 254 nm and 233 nm light sources with 40 mJ/cm^2^. Untreated skin served as control. The mean values of the epidermal DNA damage in % are calculated from at least three experiments and the error bars show the standard error of the mean. (**b**)–(**e**) Histologic images HE and CPD stained porcine skin after irradiation using the far-UVC LED irradiation system without (**c**) and with filter (**d**), in comparison, untreated skin (**e**) and skin after irradiation with near-UVC radiation at 254 nm (**f**). Arrows mark CPD positive cells. The scale bar is 100 μm.

Finally, the Supplementary Information file published with this Article contained an error in Figure S1. The original Supplementary Information file is provided below.

The original Article and Supplementary Information have been corrected.

## Supplementary Information

Supplementary Information.

