# Supplementary material for: Author Correction: Skin tolerant inactivation of multiresistant pathogens using far-UVC LEDs
Source: Sci Rep. 2022 May 11;12:7702. doi: 10.1038/s41598-022-11796-3 (PMC9095698; doi:10.1038/s41598-022-11796-3)
Supplement: Supplementary file 1 — Supplementary Information. [file 41598_2022_11796_MOESM1_ESM.docx]

Skin tolerant inactivation of multiresistant pathogens using far-UVC LEDs

Johannes Glaab^1,^ *, Neysha Lobo-Ploch^1,^ *, Hyun Kyong Cho^1^, Thomas Filler^1^, Heiko Gundlach^3^, Martin Guttmann^2^, Sylvia Hagedorn^1^, Silke B. Lohan^4^, Frank Mehnke^2, **^, Johannes Schleusener^4^, Claudia Sicher^5^, Luca Sulmoni^2^, Tim Wernicke^2^, Lucas Wittenbecher^1^, Ulrike Woggon^3^, Paula Zwicker^5^, Axel Kramer^5^, Martina C. Meinke^4^, Michael Kneissl^1,2^, Markus Weyers^1^, Ulrike Winterwerber^1^, Sven Einfeldt^1, ***^

^1^ Ferdinand-Braun-Institut, Leibniz-Institut für Höchstfrequenztechnik, Gustav-Kirchhoff-Str. 4, 12489 Berlin, Germany

^2^ Technische Universität Berlin, Institut für Festkörperphysik, Hardenbergstr. 36, 10623 Berlin, Germany

^3^ Technische Universität Berlin, Institut für Optik und Atomare Physik, Straße des 17. Juni 135, 10623 Berlin, Germany.

^4^ Center of Experimental and Applied Cutaneous Physiology, Department of Dermatology, Venerology and Allergology, Charité – Universitätsmedizin Berlin, Corporate Member of Freie Universität Berlin, Humboldt-Universität zu Berlin, Berlin Institute of Health, Charitéplatz 1, 10117 Berlin, Germany.

^5^ Universitätsmedizin Greifswald, Institut für Hygiene und Umweltmedizin, Ferdinand-Sauerbruch-Straße, 17475 Greifswald, Germany.

^*^ These authors contributed equally to this project and should be considered co-first authors.

^**^ Currently with Georgia Institute of Technology, Atlanta, GA, USA.

## ^***^ Corresponding author: phone: +49-30-6392-2630, fax: +49-30-6392-2685, e-mail: sven.einfeldt@fbh-berlin.de

## **Supplementary material**

## **Design of the cooling system**

Active cooling of the module is indispensable as the LED’s output power decreases for increased operating temperatures. With 1.25 W of dissipated power generated per LED, the irradiation system with 120 LEDs generates an average heat flux of about 2.3 W/cm^2^ over the 80 mm × 80 mm base plate. Therefore, a powerful active cooling system has been developed for the radiation unit. The LEDs are soldered on copper-core printed circuit boards (PCBs) with a 50 µm thick insulation layer. The LEDs together with this PCB technology ensure a low thermal resistance from the active region of the LED chips to the heat sink of ~ 12 K/W. A water-cooled, copper-based heat sink, with a meander shaped core, is used in the system and is maintained at a constant temperature of 18 °C. The thermal resistance of the heat sink is estimated to be ≤ 1 K/W, which leads to a total thermal resistance of ~ 13 K/W per LED and an estimated rise in temperature of the active region by only ~16 K at the maximum operation current of 100 mA. The heat generated by the LEDs during operation could warm up the surrounding air which in turn could damage the irradiated samples. Hence, a second fan-based active air cooling unit was designed to generate constant air flow in front of the LEDs.

## **Fabrication of the irradiation system**

The 120 far-UVC LEDs used in the irradiation system were mounted on 30 individual PCBs in sets of 4 LEDs. The advantage of small PCBs is that it minimizes the effort to replace LEDs that have significantly degraded during operation or have suffered catastrophic failure. In addition, the LEDs are arranged as pairs connected in series and driven by 60 separate current drivers. The current of each driver can be individually adjusted so that the spatial uniformity of the far-UVC irradiance can be optimized. The operation voltages of all the LED pairs are constantly monitored, hence, severe degradation or failure of individual LEDs can be immediately detected. A built-in dimmer allows the irradiance of the system to be adjusted to 64 levels and the irradiation duration can be set using an internal timer. To ensure that the operational temperature of the LEDs does not exceed a critical value of 56 °C detrimental to the performance of the devices, a sensor is located on the heat sink to determine the temperature and relative humidity. If the temperature exceeds an adjustable threshold value, the module automatically switches off. The measurement of the relative humidity enables the user to prevent the condensation of water on the LEDs especially under warm or humid ambient conditions.

## **Histologic images for 6-4PP stained porcine skin after far-UVC LED irradiation**


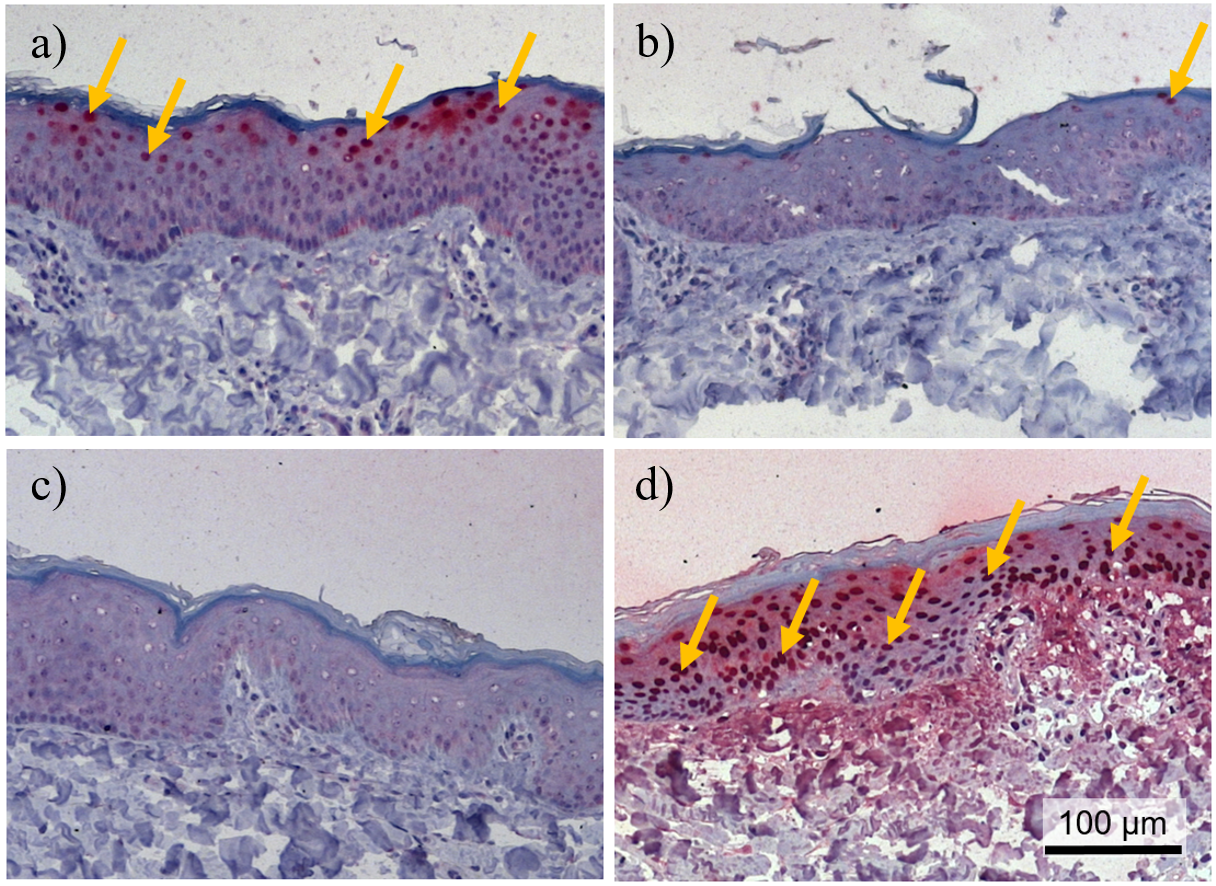


**Figure S1:** (a) – (d) Histologic images for 6-4PP stained porcine skin after irradiation using the far-UVC LED irradiation system without (a) and with filter (b), in comparison, untreated skin (c) and skin after irradiation with near-UVC radiation at 254 nm (d). Arrows mark 6-4PP positive cells. The scale bar is 100 µm.
